# Early Life Stress Interacts with the Diet Deficiency of Omega-3 Fatty Acids during the Life Course Increasing the Metabolic Vulnerability in Adult Rats

**DOI:** 10.1371/journal.pone.0062031

**Published:** 2013-04-17

**Authors:** Juliana R. Bernardi, Charles F. Ferreira, Gabrielle Senter, Rachel Krolow, Bianca W. de Aguiar, André K. Portella, Márcia Kauer-Sant'Anna, Flávio Kapczinski, Carla Dalmaz, Marcelo Z. Goldani, Patrícia P. Silveira

**Affiliations:** 1 Núcleo de Estudos da Saúde da Criança e do Adolescente (NESCA), Hospital de Clínicas de Porto Alegre (HCPA), Faculdade de Medicina, Universidade Federal do Rio Grande do Sul (UFRGS), Porto Alegre, Rio Grande do Sul, Brazil; 2 Programa de Pós-Graduação em Neurociências, Instituto de Ciências Básicas da Saúde, Universidade Federal do Rio Grande do Sul (UFRGS), Porto Alegre, Rio Grande do Sul, Brazil; 3 Departamento de Bioquímica, Instituto de Ciências Básicas da Saúde, Universidade Federal do Rio Grande do Sul (UFRGS), Porto Alegre, Rio Grande do Sul, Brazil; 4 Bipolar Disorders Program and INCT Translational Medicine (CNPq), Hospital de Clínicas de Porto Alegre (HCPA), Universidade Federal do Rio Grande do Sul (UFRGS), Porto Alegre, Rio Grande do Sul, Brazil; Max Delbrueck Center for Molecular Medicine, Germany

## Abstract

Early stress can cause metabolic disorders in adulthood. Omega-3 polyunsaturated fatty acids (n-3 PUFAs) deficiency has also been linked to the development of metabolic disorders. The aim of this study was to assess whether an early stressful event such as maternal separation interacts with the nutritional availability of n-3 PUFAs during the life course on metabolic aspects. Litters were randomized into: maternal separated (MS) and non-handled (NH). The MS group was removed from their dam for 3 hours per day and put in an incubator at 32°C on days 1° to 10° postnatal (PND). On PND 35, males were subdivided into diets that were adequate or deficient in n-3 PUFAs, and this intervention was applied during the subsequent 15 weeks. Animal's body weight and food consumption were measured weekly, and at the end of the treatment tissues were collected. MS was associated with increased food intake (*p* = 0.047) and weight gain (*p* = 0.012), but no differences were found in the NPY hypothalamic content between the groups. MS rats had also increased deposition of abdominal fat (*p*<0.001) and plasma triglycerides (*p* = 0.018) when compared to the NH group. Interactions between early life stress and n-3 PUFAs deficiency were found in plasma insulin (*p* = 0.033), HOMA index (*p* = 0.049), leptin (*p* = 0.010) and liver PEPCK expression (*p* = 0.050), in which the metabolic vulnerability in the MS group was aggravated by the n-3 PUFAs deficient diet exposure. This was associated with specific alterations in the peripheral fatty acid profile. Variations in the neonatal environment interact with nutritional aspects during the life course, such as n-3 PUFAs diet content, and persistently alter the metabolic vulnerability in adulthood.

## Introduction

The maternal separation (MS) is a well-known paradigm can be used to examine the biological consequences of early-life stress [1] [2]. The acute physiological responses to separation are well established [3] [4]. However, this model has been associated with altered mother-pup interaction, which in turn could influence the pup's development and mediate the long-term changes seen in adult offspring [5]. Regarding the consequences, maternal separated adult rats exhibit high stress responsiveness, demonstrating higher levels of glucocorticoids after acute stress and alterations in emotional and behavioral regulation when challenged in specific experimental environments [5]. They are also more vulnerable to develop a behavioral profile comparable to depression [2] [6] [7] and anxiety-like behaviors in adulthood [2] [8]. Considering the effects associated with MS, this model has been suggested to constitute an interesting model for early environmental stress and to propitiate the investigation of the long-term effects of neonatal interventions.

Stress or excess glucocorticoids exposure in sensitive phases of development such as the fetal or neonatal periods are important risk factors for the development of chronic illnesses such as obesity, type 2 diabetes mellitus, hypertension and cardiovascular disease later in life [9] [10] [11]. This has been hypothesized as a key element in the early origins of these adult diseases [10] [12] and animal models have been used to specifically investigate the mechanisms of developmental programming [13]. In monkeys, stress applied during a critical period results in the emergence of obesity and insulin resistance in the peripubertal age [14]. In rats, fetal adverse environments can prenatally affect the expression of genes that lead to the development of diabetes mellitus type 2 in the offspring [15].

Also, it is known that nutrition throughout life also impacts long-term health. Evidence shows that diets with low content of long-chain polyunsaturated fatty acids (PUFAs) are involved in the development of metabolic syndrome [16], in conjunction with altered stress responses [17], behavioral patterns [17] personality traits [18], enhanced sympathetic activity [19], increased the secretion of catecholamines [20], cortisol and serotonin [21] [22]. Epidemiological and controlled clinical studies suggest beneficial effects of n-3 PUFAs consumption, both marine and plant-derived, on cardiovascular disease [23]. However, additional studies are still necessary to elucidate all the cellular and molecular mechanisms responsible for the cardiovascular and metabolic effects of n-3 PUFAs [24].

The proposal of our study was to determine if an early stressful life event, such as a neonatal maternal separation, could interact with life-long environmental factors, such as an unbalanced nutrition, and have a long-term impact on metabolic outcomes. Our hypothesis was that early stress would increase the vulnerability to the adverse metabolic effects of a chronic n-3 PUFAs deficient diet.

## Materials and Methods

### Subjects

Pregnant Wistar rats bred at our animal facility were randomly selected. They were single-housed in home cages made of Plexiglas (65×25×15 cm) with the floor covered with sawdust and maintained in a controlled environment (lights on between 07:00 h and 19:00 h, temperature of 22 ± 2°C, cage cleaning once a week, food and water provided) until offspring. All litters were culled within 24 h to eight pups and were maintained intact unless for separation procedures, which were carried out between 15:30 h and 19:00 h. Included in this period were the time to set up the incubator, to bring the cages from the facility and briefly habituate the dams to the new room, to perform careful removal of the pups from the nest, the time of the separation per se, the return of the pups to the dam and, again after a brief period, to return the cage to the facility room. The researcher also changed gloves for the manipulation of each litter to avoid the spread of any kind of odor from nest to nest.

The day of birth was considered as postnatal day (PND) 0, and weaning was on PND 21. One or two male pups were used per litter per group per experiment. Rats were housed 2–3 per cage in home cages similar to those described above. Sixty two (n = 62) experimental male rats were used in the different experiments, derived from 12 different litters. Rats had free access to food (standard lab rat chow) and water, until PND 35, when the different diets (specially ordered for this study) were started (see below). We chose this age (PND 35) because other models have already explored the long term effect of n-3 PUFAs deficiency started soon after weaning [25] [26]. Aiming at investigating the human relevance and potential translational aspects of the study, we were interested in observing the effects of n-3 PUFAs deficiency starting during adolescence, a time in which humans begin to be more independent from their parents in terms of food choices.

### Neonatal stress model

Non-handled group (NH): pups were left undisturbed with the dam until weaning. Dirty sawdust was carefully removed from one side of the cage, without disturbing the mother and the nest, and replaced by clean sawdust at that side by the principal researcher.

Maternal separated group (MS): pups were removed from their home cage and were placed into a clean cage lined with clean paper towel, inside an incubator at 32° C. After 3 hours, pups returned to their dams. This procedure was made in the first PND 10, after which pups were left undisturbed until PND 21.

### Dietary Groups and Diets Composition

Rats were fed either adequate n-3 PUFAs (n = 29) or deficient n-3 PUFAs (n = 33) in calorically and nutritionally balanced diets, both especially manufactured for this study (PragSoluções Biociências®, São Paulo, Brazil) from PND 35 for 15 weeks [26]. Food consumption and body weight were measured weekly in digital balance (Marte®, AS2000C, São Paulo, Brazil) in all groups (n = 14 to 17 for each of the 4 groups). Chow consumption was measured for each cage and the calculation was done by mean consumption per animal per cage (n = number of cages = 29).

About the nutritional composition, each experimental diet contained 17.2 kJ/g of calories, 58.54% of carbohydrate, 19.51% of protein and 21.95% of crude fat (sources: coconut fat, canola oil and flaxseed oil). The densities of the oils were 90.5 g/100 ml for canola oil and 89.0 g/100 ml for flaxseed oil. The n-6/n-3 ratios were of 1.83 and 5.02 for the adequate and deficient diets, respectively [27] [28]. Table 1 show the composition and the detailed fatty acids contents of each diet (please find the measurements method described below).

**Table 1:** Diets composition.

Ingredients (in g)	n-3 PUFAs adequate (for 1000 g)	n-3 PUFAs deficient (for 1000 g)
Isolated Soy Protein	200	200
Dextrose	200	200
Maltodextrin	150	150
Corn starch	150	150
Sucrose	100	100
Coconut fat	60	66
Canola oil	32	34
Flaxseed oil	8	0
Microcrystalline cellulose	50	50
Mineral mix	35	35
Vitamin mix	10	10
Chlorine chlorine	2.5	2.5
L-cystine	2.5	2.5
Butylated hydroxytoluene (BHT)	0.0	0.0
**Fatty acid composition (% of the total fatty acids content)**		
8:0	1.0	1.2
10:0	1.3	1.6
12:0	21.4	23.7
14:0	11.6	12.4
15:0	0.0	0.0
16:0	8.9	9.1
16:1 n-7	0.1	0.1
17:0	0.1	0.1
18:0	4.1	3.9
18:1 n-9	30.9	31.5
18:1 n-7	1.6	1.6
18:2 n-6	11.2	10.9
20:0	0.4	0.4
18:3 n-6	0.3	0.3
20:1 n-9	0.7	0.7
18:3 n-3	6.3	2.2
20:2 n-6	0.0	0.0
24:0	0.2	0.2
24:1 n-9	0.1	0.1

### Abdominal Fat Dissection and Tissue Collection

Rats were decapitated between 13:00 h and 16:00 h in a random order using a small animal guillotine (Insight® EB271, São Paulo, Brazil) after 6 h of fasting. Trunk blood was collected and an aliquot of whole blood was stored. After centrifugation (3000 rpm, 15 min), serum and plasma were also stored at −80°C until glucose, total cholesterol, triglycerides (TGs), insulin and leptin determination. A small portion of the liver, as well as the whole brain were quickly removed, flash frozen in isopentine and the samples were stored at −80°C until processing. The two major portions of abdominal fat (gonadal and retroperitoneal adipose tissue depots) were dissected and weighed.

### Detailed dietary fatty acid composition and peripheral blood fatty acids determination

Dietary fat was extracted with chloroform-methanol [29]. Aliquots (2–3 ml) of the chloroform lipid extract were evaporated at 50°C using a vacuum pump, followed by saponification in methanolic KOH solution and esterification in methanolic H2SO4 solution [30].

Fatty acids in blood were esterified and extracted using a one-step reaction [31] with some modification. Blood (100 µl) was mixed with 2 ml methanol/isooctane (4∶1, v/v) and 200 µl acetyl chloride and was incubated at 100°C for 60 min; then 60 g/l aqueous potassium carbonate containing 100 g/l sodium chloride was added. The mixture was shaken for 10 min at room temperature and centrifuged at 1,800 g for 5 min, to obtain the isooctane phase, containing the fatty acid methyl esters.

The esterified samples were analyzed using an Agilent Technologies gas chromatograph (HP 6890) fitted with a Supelco SP-2560 capillary column (100 m×0.25 mm×0.20 µm) and flame ionization detection. The temperature of the injector port and the detector was set at 250°C, and the carrier gas was nitrogen (1.0 ml/min). After injection (1 µl, split ratio 50∶1), the oven temperature was hold at 140°C for 3 min, increased to 170°C at a rate of 6°C min−1, and hold at this temperature for 2 min, increased to 185°C at a rate of 3°C min-1, and hold at this temperature for 6 min, and then increased to 240°C at a rate of 3°C min-1, and hold at this temperature for 7 min. Standard fatty acid methyl esters (37-component FAME Mix, DPA n-3 and PUFA no. 2 from Sigma, Saint Louis, MO, USA and DPA n-6 from NuChek Prep. Inc., Elysian, MN, USA) were run under the same conditions and the subsequent retention times were used to identify the fatty acids. Fatty acids were expressed as percentage of the total fatty acids content.

### Biochemical Analysis

The biochemical parameters were measured in a representative subsample of 30 rats because after the 15 weeks of the exposure to the diets, the other animals (n = 32) were subjected to behavioral tasks planned in the project but not related to metabolic outcomes reported in this study. As behavioral testing could affect the metabolic results, we decided not to include these animals in the biochemical measurements. Plasma glucose, plasma cholesterol (total and HDL, LDL was calculated) and plasma TGs were measured using commercial kits (Wiener lab Group®, São Paulo, Brazil) according to the manufacturer's instructions. Serum insulin and serum leptin were measured using commercially available ELISA kits (Millipore®, EZRMI-13K, Missouri, USA and Invitrogen®, KRC2281, California, USA, respectively). The HOMA index was calculated as the product of the fasting plasma insulin and glucose levels divided by 22.5 [32]. The whole hypothalamus was dissected from the brain at −20°C, the samples were homogenized in Nuclear Extraction Buffer I (NEB1–10 mM HEPES, 10 mM KCl, 0.1 mM EDTA, 0.1 mM EGTA, pH 7.9) with a protease inhibitor (100∶1). Then, a detergent (NP40 1%) was added and the homogenate was centrifuged at 6000 rpm for 10 minutes at 4°C. The supernatant (cytosolic fraction) was used for posterior NPY quantification using a commercially available peptide enzyme immunoassay kit (Peninsula Laboratories LCC, California, USA).

### Real-time quantitative PCR

For gene expression assays, PECK expression, total RNA was extracted from liver using TRI Reagent (Invitrogen) according to the manufacturer's instructions. RNA concentrations were measured with NanoDrop® ND-1000 (NanoDrop Technologies, Montchanin, DE, USA). It is well established that the ratios of absorbencies at 260 nm and 280 nm and 260 nm and 230 nm is used to assess the purity of RNA. All samples were analyzed and the ratios approximately at 2.0 and 2.0–2.2, respectively, were accepted as pure RNA. The selection of GAPDH as an endogenous control was based on several studies that demonstrate the efficiency of its expression in rat liver [33] [34] [35]. Although a pool of different internal controls wasn’t performed, the GAPDH expression did not vary between the samples tested, justifying its use.

All reactions were performed in triplicates with the 7500 Real Time PCR System (Applied Biosystems). One microgram of total RNA was reverse-transcribed to cDNA using High Capacity cDNA Reverse Transcription Kit (P/N: 4368814, Applied Biosystems, CA, USA) following the manufacturer's protocol and cDNA was stored at −80°C until real-time quantitative reverse transcription-polymerase chain reaction (RT-PCR) analysis. All cDNAs were diluted 1∶10 before being used as PCR template. The dilution was chosen after standard curve was performed. Specific primer pairs and TaqMan MGB probe labeled with FAM for Phosphenolpyruvate carboxykinase (PEPCK) (Rn 01529014_m1, Applied Biosystems) were used. Glyceraldehyde-3-phosphate dehydrogenase (GAPDH) primers and probe (43523388E, Applied Biosystems) labeled with VIC were used as endogenous control. Relative expression levels were determined by the ddCt method [36].

### Statistical Analyses

Data were expressed as Mean values ± SEM (Standard Error of the Mean) and analyzed by Student's t Test, two-way or repeated measures analysis of variance (ANOVA), followed by LSD *post hoc* test, when indicated. Data on PEPCK gene expression had no normal distribution and therefore it was log transformed for analysis. Pearson correlations were performed between the different fatty acids levels and different outcomes. Significance levels for all measures were set at *p*≤0.05. SPSS (Statistical Package for the Social Sciences®; SPSS Inc, Chicago, IL) version 18.0 was used for the statistical analysis.

### Ethical Aspects

All animal treatments were approved by the Institutional Ethical Committee (Ethical Committee, Universidade Federal do Rio Grande do Sul, with number 09-410) and followed the recommendations of the International Council for Laboratory Animal Science (ICLAS). All efforts were taken to minimize pain or discomfort.

## Results

### Body Weight Gain and Food Consumption

**Figure 1:**
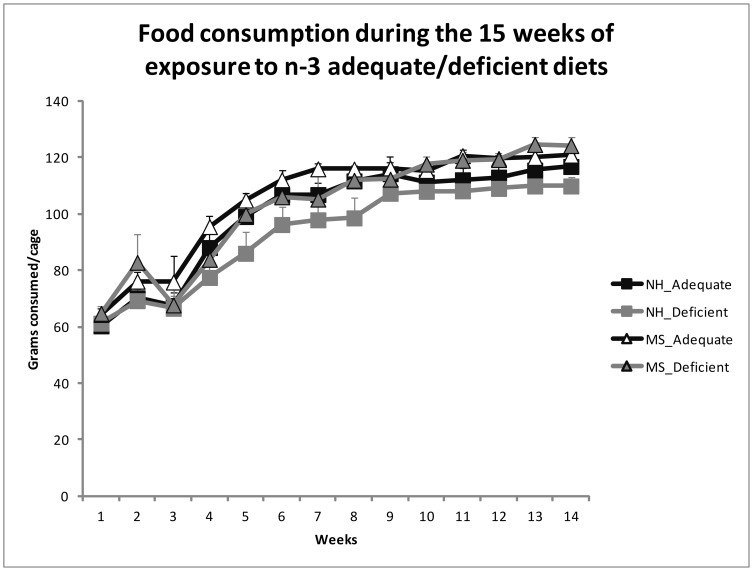
Mean ± SEM of the weekly chow consumption (mean consumption per cage) during the 15 weeks period of chronic exposure to the diets (from PND 35). Maternal separated (MS) rats had increased food consumption in comparison to the non-handled (NH) ones (Repeated Measures ANOVA, *p* = 0.047). There was no effect of the diets on the food consumption (*p* = 0.245), or interaction (MS_ adequate group = 6 cages; MS_ deficient group = 6 cages; NH_ adequate group = 7 cages; NH_ deficient group = 10 cages).

**Figure 2:**
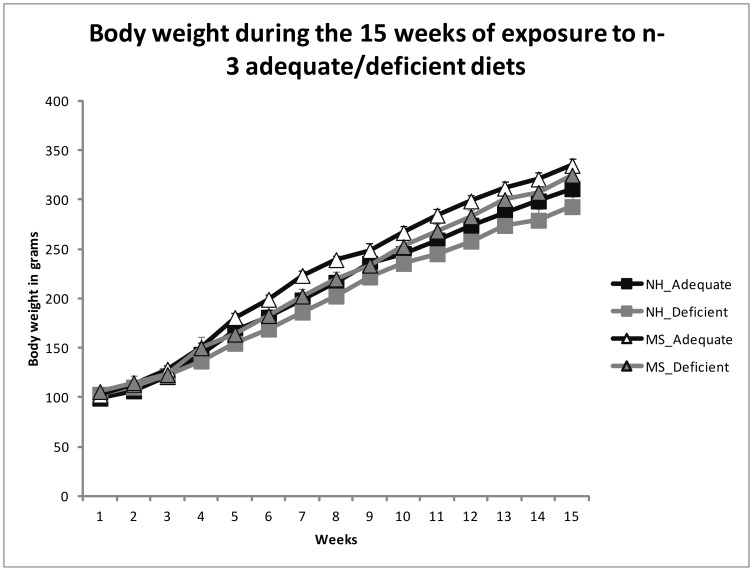
Mean ± SEM of the weekly body weight measurements during the 15 weeks period of chronic exposure to the diets (from PND 35). Maternal separated (MS) rats had increased body weight in comparison to the non-handled (NH) ones (Repeated Measures ANOVA, *p* = 0.012). There was no effect of the diets on the body weight (*p* = 0.099), or interaction.

MS was associated with increased body weight already at ages PND 21 (NH 36.09±1.08; MS 40.07±0.96), PND 28 (NH 65.60±1.71; MS 70.92±1.43) and PND 35 (NH 105.90±2.30; MS 112.81±1.83), before the exposure to the adequate or deficient diets (Student's t Test, t(60) = 2.742, *p* = 0.008; t(60) = 2.366, *p* = 0.021 and t(60) = 2.327, *p* = 0.023, respectively). There was an effect of group, where maternal separated (MS) rats had more weight gain [Repeated Measures ANOVA, F(1,58) = 6.719, *p* = 0.012] and food consumption [F(1,25) = 4.345, *p* = 0.047] than the NH group, without interactions [F(1,58) = 0.009, *p* = 0.926 for weight, F(1,25) = 0.273, *p* = 0.606 for food intake]. It is important to mention for comparison that in our animal facility, the mean body weight of 5 month old Wistar rats fed with standard chow attain between 330 g and 370 g. There were no diet effects in body weight [F(1,58) = 2.815, *p* = 0.099] or food consumption [F(1,25) = 1.418, *p* = 0.245]. Figures 1 and 2 display these results, respectively.

### Abdominal Fat

**Figure 3:**
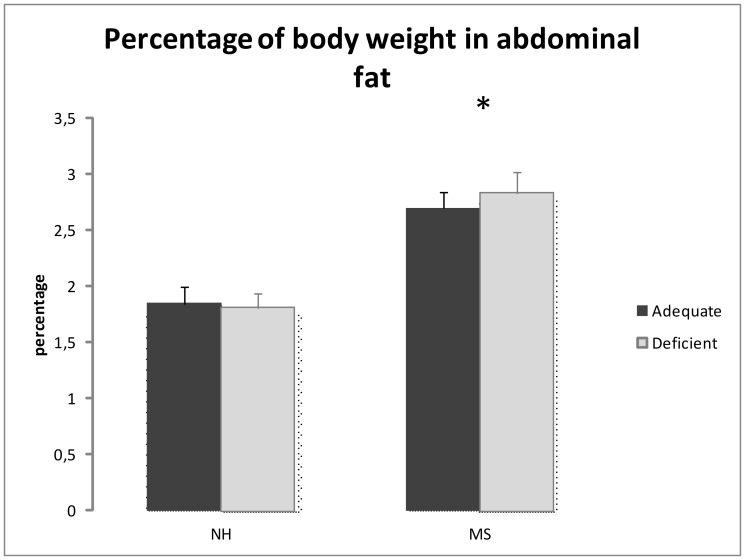
Abdominal fat [(sum of gonadal and retroperitoneal depots' weight in grams)*100/total body weight in grams)]. Maternal separated (MS) rats had increased abdominal fat deposition in comparison to the non-handled (NH) ones (*Two-Way ANOVA, *p*<0.001] without effect of the diets (*p* = 0.756) or interaction.

Abdominal fat deposition is expressed in percentage of body weight of each rat. The MS group had more abdominal fat deposition [Two-Way ANOVA, F(1,61) = 35.378, *p*<0.001] than NH group, without effect of the diets [F(1,61) = 0.097, *p* = 0.756] or interaction [F(1,61) = 0.327, *p* = 0.570]. Figure 3 displays these results.

### Biochemical Analysis

Results from biochemical measurements are shown in Table 2. The MS group had increased plasma triglycerides [Two-Way ANOVA, F(1,29) = 6.403, *p* = 0.018] when compared to the NH group, without effect of the diet [F(1,29) = 0.707, *p* = 0.408] and no interaction [F(1,29) = 1.212, *p* = 0.281]. There was no difference in plasma glucose [F(1,29) = 1.071, *p* = 0.310], total cholesterol [F(1,29) = 2.142, *p* = 0.155], LDL [F(1,28) = 3.731, *p* = 0.065] and HDL [F(1,28) = 0.022, *p* = 0.882] between groups and no effects of the diet [F(1,29) = 0.473, *p* = 0.497; F(1,29) = 2.895, *p* = 0.101, F(1,28) = 0.768, *p* = 0.389 and F(1,28) = 0.008, *p* = 0.931 respectively], and no interactions [F(1,29) = 0.151, *p* = 0.701; F(1,29) = 0.686, *p* = 0.415; F(1,28) = 0.024, *p* = 0.878 and F(1,28) = 0.011, *p* = 0.917 respectively]. The hypothalamic content of NPY was also no different among the groups [F(1,24) = 0.288, *p* = 0.597] and diets [F(1,24) = 0.022, *p* = 0.884], with no interactions [F(1,24) = 0.433, *p* = 0.518].

**Table 2:** Mean ± SEM for biochemical measurements.

Groups	Non-handled		Maternal Separation	
Diets	Adequate n = 7	Deficient n = 8	Adequate n = 7	Deficient n = 8
Glucose (mg%)	100.7±4.5	99.5±4.0	106.5±4.2	102.1±3.6
Total cholesterol (mg%)	60.9±1.8	63.3±2.9	54.8±2.0	61.6±3.2
HDL (mg/dl)	25.5±1.5	24.7±2.6	24.6±3.5	24.6±4.8
LDL (mg/dl)	21.5±2.8	26.1±3.7	13.8±2.4	16.9±6.5
Triglycerides (%mg)*	69.6±5.2	67.2±5.8	82.5±11.3	99.9±11.5
Insulin (ng/mL)**	1.8±0.1^a,b^	1.6±0.2^b^	1.4±0.3^b^	2.6±0.5^a^
Leptin (pg/mL)**	2442.6±379.4^b^	1445.1±115.8^b^	2819.1±265.2^a,b^	3541.4±365.8^a^
HOMA index**	0.5±0.1^a,b^	0.4±0.1^b^	0.4±0.1^b^	0.7±0.1^a^
Liver PEPCK mRNA(% RQ values of NH_adequate group)**	100.0±9.0	78.9±9.0	75.7±9.8	89.5±7.8
Hypothalamic NPY(ng/mg)	1.5±0.1	1.6±0.2	1.7±0.3	1.6±0.1

Different letters mean statistical significant differences in the multiple comparisons (LSD test).

* Two-Way ANOVA: Effect of the Maternal Separation, p< = 0.05.

** Two-Way ANOVA: Interaction Maternal Separation x Diet, p< = 0.05.

There was an interaction between the MS group and the diet exposure in serum insulin [F(1,29) = 5.083, *p* = 0.033], in which MS_deficient animals were different from MS_adequate (*post hoc* LSD, *p* = 0.013) and NH_deficient (*p* = 0.022) whereas the difference from NH_adequate did not reach statistical significance (*p* = 0.078). An interaction was also seen for HOMA index [F(1,29) = 4.276, *p* = 0.049], in which the pattern is similar (*p* = 0.023, *p* = 0.022 and *p* = 0.075 for the difference between MS_deficient and MS_adequate, NH_deficient and NH_adequate, respectively). No isolated effects of group [F(1,29) = 1.143, *p* = 0.295 for insulin; F(1,29) = 1.619; *p* = 0.214 for HOMA index] or diet [F(1,29) = 2.320, *p* = 0.140 for insulin; F(1,29) = 1.816, *p* = 0.189 for HOMA index] were found for these outcomes.

Serum leptin [F(1,28) = 7.840, *p* = 0.010] also demonstrated an interaction, in which MS_deficient animals were different from NH_adequate (*post hoc* LSD, *p* = 0.037) and NH_deficient (*p* = 0.001) whereas the difference from MS_adequate did not reach statistical significance (*p* = 0.160). Liver PEPCK gene expression followed the same pattern of interaction [F(1,24) = 4.239, *p* = 0.050], but the multiple comparisons was not able to detect the differences. No isolated effects of diet [F(1,28) = 0.202, *p* = 0.657 for leptin and F(1,24) = 0.114, *p* = 0.739 for PEPCK] was found for these outcomes, and while no isolated effect of the group was found for PEPCK [F(1,24) = 0.509, *p* = 0.484] an effect of group on leptin levels [F(1,28) = 16.175, *p*<0.0001], which disappears after adjusting the ANOVA for the abdominal fat deposition (% of body weight), while the interaction group x diet remains significative [F(1,28) = 0.923, *p* = 0.346 for group; F(1,28) = 0.558, *p* = 0.462 for diet and F(1,28) = 5.416, *p* = 0.029 for the interaction]. These results were displayed in Table 2.

### Peripheral blood fatty acids determination

Two ANOVAs were performed to analyze the peripheral blood fatty acids. There was an interaction between the MS group and the diet in the peripheral levels of the 16∶1 n-7 fatty acid [F(1,16) = 5.703, *p* = 0.030], in which MS_deficient rats had increased levels in comparison to NH_deficient rats (*post hoc* LSD, *p* = 0.034). There were no isolated effects of group [F(1,16) = 0.802, *p* = 0.384] or diet [F(1,16) = 0.003, *p* = 0.957] for this fatty acid.

An interaction was also seen in the peripheral levels of the 18∶3 n-3 fatty acid [F(1,16) = 5.828, *p* = 0.028], in which NH_deficient rats had decreased levels when compared to NH_adequate (*post hoc* LSD, *p* = 0.007), but also when compared to MS_deficient (*post hoc* LSD, *p* = 0.044) rats; the other group comparisons were not significant. There were no isolated effects of group [F(1,16) = 0.450, *p* = 0.512] or diet [F(1,16) = 3.664, *p* = 0.074] for this fatty acid.

An isolated effect of the diet was seen in the peripheral levels of the 22∶6 n-3 fatty acid (DHA) [F(1,16) = 5.161, *p* = 0.037], sum of saturated fatty acid [F(1,16) = 4.594, *p* = 0.048], and total n-6 PUFAs [F(1,16) = 14.707, *p* = 0.001], in which rats receiving the deficient diet had increased peripheral DHA levels and total n-6 PUFAS and decreased saturated fatty acids. There were no isolated effects of group in DHA [F(1,16) = 0.980, *p* = 0.337]; saturated fatty acids [F(1,16) = 1.000, *p* = 0.332] and n-6 PUFAs [F(1,16) = 0.617, *p* = 0.443]; or interaction in DHA [F(1,16) = 1.328, *p* = 0.266]; saturated fatty acids [F(1,16) = 1.612, *p* = 0.222] and n-6 PUFAs [F(1,16) = 0.000, *p* = 0.999]; for this fatty acid. Despite that, there were no differences in total n-3 PUFAs [F(1,16) = 0.111 *p* = 0.744 for group, F(1,16) = 0.782, *p* = 0.390 for diet and F(1,16) = 0.385, *p* = 0.544 for the interaction] or in the omega 3 index (EPA-Eicosapentaenoic acid + DHA) [F(1,16) = 0.537, *p* = 0.537 for group, F(1,16) = 0.245, *p* = 0.627 for diet and F(1,16) = 0.270, *p* = 0.871 for the interaction] between the groups.

No other differences or isolated effects were observed (Table 3). However, negative correlations between the peripheral levels of 18∶0 (stearic acid) and HOMA index (r = −0.553, *p* = 0.014), insulin (r = −0.920, *p* = 0.027) and triglycerides levels (r = −0.580, *p* = 0.009). Besides that, the peripheral levels of 16∶1 n-7 (palmitoleic acid) correlate positively with the abdominal fat deposition (as a percent of body weight) (r = 0.486, *p* = 0.035).

**Table 3:** Mean ± SEM peripheral blood fatty acid composition in non-handled and maternal separated male rats fed adequate or n-3 PUFAs deficient diets.

Group Diets	Non-handled		Maternal Separated		*p* Value		
	Adequate (mg/100 mg fatty acid)	Deficient (mg/100 mg fatty acid)	Adequate (mg/100 mg fatty acid)	Deficient (mg/100 mg fatty acid)	Group	Diet	Interaction
12∶0	0.5±0.6	0.4±0.4	1.1±0.4	0.9±0.7	0.060	0.539	0.788
14∶0	0.8±0.6	0.5±0.4	0.6±0.1	0.6±0.2	0.563	0.388	0.474
16∶0	24.2±2.1	25.0±0.3	24.2±2.0	23.1±1.4	0.209	0.846	0.181
16∶1 n-7	0.4±0.5^a,b^	0.0±0.0^b^	0.2±0.3^a,b^	0.6±0.6^a^	0.384	0.957	0.030
17∶0	0.2±0.3	0.4±0.4	0.2±0.3	0.2±0.3	0.369	0.369	0.640
18∶0	18.3±2.2	18.2±0.9	19.1±1.7	16.8±2.5	0.708	0.178	0.210
18∶1 n-9	13.9±2.0	14.0±0.6	15.2±1.7	16.3±3.1	0.063	0.527	0.551
18∶1 n-7	1.7±0.5	1.9±0.2	1.9±0.4	2.2±0.3	0.213	0.142	0.713
18∶2 n-6	11.1±1.6	11.7±1.6	11.3±1.5	13.6±2.5	0.206	0.092	0.311
20∶1 n-9	0.1±0.2	0.3±0.5	0.1±0.1	0.2±0.3	0.620	0.164	0.894
18∶3 n-3	0.8±0.3^b^	0.1±0.2^a^	0.5±0.4^a,b^	0.6±0.5^b^	0.512	0.074	0.028
22∶0	0.7±0.7	0.2±0.5	0.4±0.5	0.3±0.5	0.723	0.375	0.404
20∶3 n-6	1.0±0.8	1.2±0.8	0.7±0.7	0.7±0.7	0.261	0.759	0.791
20∶3 n-3	0.2±0.4	0.1±0.3	0.3±0.5	0.0±0.0	0.975	0.264	0.431
20∶4 n-6	11.8±1.0	13.9±1.4	11.3±2.1	11.8±3.0	0.174	0.172	0.405
24∶0	3.8±1.9	2.6±1.7	3.3±1.9	2.9±1.8	0.906	0.352	0.620
20∶5 n-3	1.2±0.8	0.0±0.0	0.7±1.0	0.6±0.9	0.909	0.075	0.112
24∶1 n-9	3.7±1.2	3.3±0.4	3.5±1.0	2.7±1.4	0.397	0.173	0.723
22∶5 n-3	1.7+0.4	1.0+0.7	1.5+0.9	1.1+0.8	0.935	0.099	0.605
22∶6 n-3	2.7+0.6^a^	4.1+1.6^b^	2.8+0.4^a^	3.2+0.7^b^	0.337	0.037	0.266
Saturated	48.5+3.5^a^	47.4+1.2 ^b^	48.8+2.7^a^	44.7+2.8^b^	0.332	0.048	0.222
Monounsaturated	20.0+2.4	19.5+0.8	20.8+1.1	22.0+2.7	0.065	0.680	0.335
n-3 PUFAs	6.7+1.4	5.4+2.0	5.8+2.0	5.6+2.2	0.744	0.390	0.544
n-6 PUFAs	23.9+1.9^a^	26.8+0.9^b^	23.4+2.2^a^	26.2+1.4^b^	0.443	0.001	0.999
n-6/-n-3 ratio	3.7+0.6	5.6+2.0	4.5+1.9	5.4+2.4	0.734	0.112	0.576

Different letters mean statistical significant differences in the multiple comparisons (LSD) test.

## Discussion

In this study, maternal separation stress was associated with increased weight gain, food consumption, abdominal fat and plasma triglycerides in adult rats. In addition, the chronic exposure to a mild deficiency in dietary n-3 PUFAs potentiates the vulnerability conferred by the neonatal stress, leading to insulin resistance and altered leptin, as well as specific alterations in the peripheral fatty acids profile.

We showed that maternal separation stress is associated with higher body weight that can be detected at weaning (before the exposure to the dietary deficiency in n-3 PUFAs), as well as increased food intake and abdominal fat, irrespective to the diet treatment. Interestingly, the food intake pattern from week 1 to 3 is broken in all groups by a sudden increased food intake during the second week of the diet exposure. Others have shown a similar irregularity during the first food intake measurements when changing the type of diet and/or the dietary fat content [37], and this is possibly related to the rats' adaptation to the new food.

Regarding the early intervention model, studies suggests that neonatal maternal separation can lead to the development of eating disorders, especially when the animal is challenged with other social or metabolic stressors later in life [2]. The resulting positive energy balance, especially in an animal known to have a maladaptive response to stress [5], with amplification of glucocorticoid action, is probably playing an important role in the development of the central abdominal obesity [38] that we described in this group. The increased abdominal fat is suggested as a marker of 'dysfunctional adipose tissue' [39], leading to altered free fatty acids metabolism, and hypertrigliceridemia as we saw in maternal separated animals. The resulting increased free fatty acids flux to the liver impairs liver metabolism [40], leading to increased hepatic insulin resistance [41]. As n-3 PUFAs are potent inhibitors of hepatic glycolysis and de novo lipogenesis, through the inhibition of genes involved in glucose utilization and lipid synthesis, including L-pyruvate kinase, fatty acid synthase, and acetyl-CoA carboxylase [42], a dietary deficiency in this nutrient would exacerbate hepatic fatty acid synthesis and storage [43], and therefore liver lipotoxicity [40].

The increased hepatic insulin resistance decreases the ability of insulin to downregulate PEPCK gene expression as we saw in the MS, n-3 PUFAs deficient rats [44] [45]. This impairs the shutdown of liver glucose production and consequently increases the need of insulin production to maintain a normal glycemia. We could consider this “effort” as an indicative of increased allostatic load in this group [46].

As insulin is one of the major regulators of leptin levels [47] [48], the increased peripheral insulin reported here could explain the altered levels of leptin described in maternally separated, n-3 PUFAs deficient rats. Strikingly, despite the higher circulating levels of the anorexigenic cytokine leptin, maternal separated rats still had increased food intake, which was also not explained by differences in hypothalamic NPY (a neuropeptide that plays a major role stimulating appetite). Another study reports an increased NPY mRNA level only after 48 h of food deprivation in MS females, but no differences in the basal arcuate expression levels of this peptide [49]. This result agrees with our study, in the sense that hypothalamic NPY levels do not appear to respond to peripheral signals of energy homeostasis in MS animals in the same way that NH animals would do. Since leptin is known to decrease NPY release [50], the absence of effect on this peptide levels could be interpreted as a sign of hypothalamic leptin resistance in the maternally separated, n-3 PUFAs deficient rats.

A study evaluating the effects of dietary flaxseed, a lipid rich in n-3 PUFAs showed no difference in weight gain, food consumption and caloric efficiency in rats. However, there was significant decrease in blood glucose, total cholesterol and triglycerides in animals that received flaxseed when compared to animals that received standard food [51]. Our study did not show differences in blood glucose or cholesterol, while triglycerides were affected mainly by the neonatal intervention. Other studies describe that a dietary deprivation of n-3 PUFAs for no more than 3 months is sufficient to cause a severe depletion of these fatty acids in both liver phospholipids and triglycerides [52] and induces features of the metabolic syndrome, including liver steatosis, visceral obesity and insulin resistance [53] [54]. In our study, these effects were seen only in neonatally stressed rats, not in NH group. In other words, maternal separation conferred an increased vulnerability to the metabolic effects of the dietary n-3 PUFAs deficiency in our study.

Other authors have reported that the MS procedure leads to a significant reduction in total plasma n-3 PUFAs concentrations (including α-linolenic acid, eicosapentaenoic acid, docosapentaenoic acid and docosahexaenoic acid) in comparison to controls. We did not find differences in the peripheral levels of n-3 PUFAs in our study, and the deficient diet induced a reduction in peripheral α-linolenic acid only in NH animals. It is possible that the MS group was already functioning at its lowest biological limit (assuming that the lowest biological limit is higher for the MS group), and was unresponsive to the deficiency in our study. Another possibility is that we are observing more the effects of the early-life stress itself, rather than a diet X stress interaction *per se* in this result.

Interestingly, animals exposed to MS and to the dietary deficiency in n-3 PUFAs demonstrated increased palmitoleic acid in our study. Palmitoleic acid is associated with increased insulin concentrations [55] and resistance [56] [57] [58] [59] [60] [61] [62], type II diabetes [63] [64], metabolic syndrome [61] [65] [66], heart failure [67] and coronary heart disease [68]. Plasma palmitoleic acid content is an independent marker of both triglyceridemia and abdominal adiposity in men [62] [69], which agrees with the positive correlation between this fatty acid peripheral level and the amount of abdominal fat deposition found in this study. In addition, it has been shown to induce hepatic steatosis and increase fatty acid synthase expression in mice [70], corroborating to the idea of hepatic lipotoxicity in MS_deficient animals, as discussed above. Our findings also agree with the literature regarding the negative correlations between stearic acid and HOMA index as well as insulin levels [58] [63].

Despite the fact that the n-3 PUFAs deficient diet used in this study ultimately increased the peripheral levels of DHA, the groups had no difference in other n-3 PUFAs indices such as the Omega-3 Index and total n-3 PUFAs in blood. One possible explanation for the increased peripheral DHA levels is based on the very mild deficiency imposed by the diet that we used. A study using different dietary content of α-linolenic acid demonstrates that, at such level of deficiency, DHA in plasma was not affected by the diet [71]. Another study shows that DHA levels decrease more slowly than EPA subsequent to n-3 PUFAs reduction, indicating some type of docosahexaenoic acid retention [72]. In the serum, the effects of a low n-3 PUFAs diet are less severe than in other tissues [73]. In addition, it seems that the blood compartmental metabolism of DHA differs substantially depending on the type of the diet, both in terms of bioavailability in plasma and accumulation in target tissues [74]. As plasma and erythrocyte EPA, DHA, Omega-3 Index and total n-3 PUFAs have weaker correlations with the corresponding fatty acid content in the brain compared with other tissues [75], the animals receiving the deficient diet in our study could still be experiencing low levels of essential n-3 PUFAs in target tissues, especially the brain, and this could explain the metabolic effects seen especially in the MS group. Finally, our deficient diet is more closely related to mild n-3 PUFAs deficiencies that can be easily found in human populations, therefore one could argue that our results have the potential of being more readily translatable to the human conditions. The modern Western diet can reach n-6/n-3 ratio as high as 30∶1, when the desirable vary between 2∶1 to 5∶1 [76]. The ratio in our deficient diet was 5∶1 - again, a mild deficiency. This is of added significance to our findings, considering that possibly the interactions between early life stress and nutritional deficiency in n-3 PUFAs could have impacted even more the metabolic outcomes if the dietary deficiency imposed were to be more intense. It remains to be established to what extend our findings are applicable to humans, however the fact that any adult could be experiencing the same or worse levels of dietary deficiency as the one imposed by our model, is certainly of interest. It is interesting to highlight that neonatal stress interacted with the chronic n-3 PUFAs deficiency increasing the metabolic vulnerability. Other reports using a similar design demonstrated that maternal separation interacts with dietary n-3 PUFAs deprivation increasing the depressive-like response, reward response and impulsivity [26], being associated with more anxiety and fearful responses in inescapable situations, while their ability to cope with an aversive avoidance task remains unaffected [25]. In humans, several studies show that metabolic syndrome often is co morbid with major depression (just to cite recent ones: [77] [78] in a bidirectional association [79]).

Recently, a resting-state functional magnetic resonance brain imaging study [80] demonstrated that alterations in the ventral striatum connectivity mediate the relationship between HOMA index and symptoms of depressed mood. This study supports the notion that functional alterations in reward circuits vary directly with the insulin resistance levels across individuals. In addition, the role of n-3 PUFAs in mood disorders has been extensively (reviews in [81] [82] [83]). Therefore, it is possible that the insulin resistance reported in our study, in maternally separated/n-3 PUFAs deficient rats, plays a role in explaining the behavioral alterations described by [26]. It would be interesting to determine whether a diet supplemented with n-3 PUFAs might have a protective influence in MS rats, therefore future studies are warranted.

In summary, we showed that maternal separation leads to increased food intake and weight gain and its related metabolic consequences such as abdominal fat deposition and increased triglycerides. To the best of our knowledge, this is the first report describing the metabolic consequences of disruptions in the mother-pup interaction using this model. In humans, it was recently shown that the risk for overweight at age 9–11 years is elevated among children whose mothers had experienced distress in the postnatal period [84]. In addition, we observed here that a mild dietary deficiency in n-3 PUFAs increases some of the metabolic consequences of maternal separation, leading to insulin and leptin resistance, and this is associated with alterations in the peripheral fatty acid profile in MS_deficient animals, such as increased palmitoleic acid. Studies addressing if variations in the vulnerability to subtle nutritional variations also exist in humans may be relevant for primary prevention of prevalent conditions such as obesity and the metabolic syndrome, especially in populations exposed to early life stress.
